# Associations of sleep characteristics with all-cause, cardiovascular and non-cardiovascular mortality among rural Chinese older adults: a cohort study

**DOI:** 10.1136/bmjopen-2024-094928

**Published:** 2025-06-22

**Authors:** Jie Lu, Rui Liu, Jiafeng Wang, Keke Liu, Juan Ren, Tong Zhao, Yifei Ren, Ming Mao, Yajun Liang, Shi Tang, Yifeng Du, Chengxuan Qiu

**Affiliations:** 1 Department of Neurology, Shandong Provincial Hospital Affiliated to Shandong First Medical University, Jinan, Shandong, China; 2 Department of Ultrasound, Shandong Provincial Hospital Affiliated to Shandong First Medical University, Jinan, Shandong, China; 3 Department of Neurology, Shandong Provincial Hospital, Shandong University, Jinan, Shandong, China; 4 Shandong Provincial Clinical Research Center for Neurological Diseases, Jinan, China; 5 Department of Global Public Health, Karolinska Institutet, Stockholm, Sweden; 6 Institute of Brain Science and Brain-Inspired Research, Shandong First Medical University & Shandong Academy of Medical Sciences, Jinan, China; 7 Aging Research Center, Department of Neurobiology, Care Science and Society, Karolinska Institutet-Stockholm University, Stockholm, Sweden

**Keywords:** Mortality, Cardiovascular Disease, Aged

## Abstract

**Abstract:**

**Study objectives:**

The longitudinal associations of sleep timing and time in bed (TIB) with all-cause, cardiovascular and non-cardiovascular mortality are unclear in Chinese rural populations.

**Methods:**

This population-based cohort study included 2468 participants who were aged ≥60 years and residing in rural communities in western Shandong Province. Sleep timing and TIB were assessed using standard questionnaires at baseline in 2014. Mid-sleep time was defined as the halfway point between the bedtime and wake-up time. Vital status until December 2022 and causes of death for all participants were ascertained via death registry plus interviews with informants (eg, family members or village doctors). Data were analysed using restricted cubic splines (RCS) and Cox proportional-hazards models.

**Results:**

During the mean follow-up of 7.36 (SD 2.03) years, 657 participants died. The RCS analysis showed non-linear relationships of sleep duration and mid-sleep time at baseline with all-cause and cardiovascular mortality. Specifically, when baseline sleep characteristics were categorised into tertiles, the multivariable-adjusted HR for all-cause mortality was higher for long sleep duration (>8 vs 7–8 hours; HR 1.27; 95% CI 1.06 to 1.53), long TIB (>9 vs <8 hours; 1.63; 1.27 to 2.08), early bedtime (before 21:00 vs 22:00 or later; 1.58; 1.00 to 2.49) and early mid-sleep time (before 01:00 vs 01:00 –01:30; 1.45; 1.20 to 1.76). Long TIB was associated with a multivariable-adjusted HR of 1.61 (1.15 to 2.27) for cardiovascular mortality and 1.64 (1.09 to 2.47) for non-cardiovascular mortality.

**Conclusions:**

Long sleep duration and early sleep timing might be associated with increased risk of all-cause and cardiovascular mortality in rural Chinese older adults. In addition, long TIB might be linked to an increased risk of all-cause, cardiovascular and non-cardiovascular mortality.

**STRENGTHS AND LIMITATIONS OF THIS STUDY**

- The strengths of our study include a population-based design involving rural-dwelling older adults in China and the assessments of multiple sleep characteristics.
- The self-reported sleep characteristics may be subject to recall bias.
- We only examined baseline sleep characteristics, which may change over the follow-up period.
- Despite adjustment for multiple potential confounders, residual confounding may still exist.

## Introduction

 Sleep is essential for human health, with individuals typically spending about a third of their lives asleep. Sleep has been increasingly recognised as an important lifestyle behaviour that can affect cardiovascular health and mortality.^1^,^4^ A meta-analysis of prospective cohort studies suggested that extreme sleep duration and poor sleep quality were associated with increased risks of cardiovascular disease (CVD) and mortality.^5^ However, the association of time in bed (TIB), a composite indicator of sleep duration, latency and fragmentation, with mortality in older adults is poorly understood. In addition, sleep timing, a behaviour marker of circadian rhythms,^6^ has been associated with diabetes and hypertension.^7 8^ However, there is a scarcity of research on the temporal relationships between sleep timing and the subsequent risk of all-cause and cause-specific mortality in older adults.

Furthermore, several cohort studies have linked sleep duration with mortality among adults in the USA,^9 10^ Japan^11^ and Spain,^12^ whereas their association among rural older adults has not yet been well characterised. This is important because, compared with urban residents in Western countries, rural-dwelling older adults in China have distinct sleep profiles (eg, usually go to bed earlier, rise earlier and have poorer sleep)^13^ and the association between long sleep duration and mortality appears to be stronger in East Asian populations than in North American or European populations,^14^ partly due to differences in socio-economic status, culture and lifestyles.

Therefore, in this population-based cohort study of rural-dwelling Chinese older adults, we sought to examine the associations of self-reported sleep characteristics (eg, sleep duration, TIB, sleep timing and sleep quality) with all-cause, cardiovascular and non-cardiovascular mortality.

## Methods

### Study design and participants

**Figure 1:**
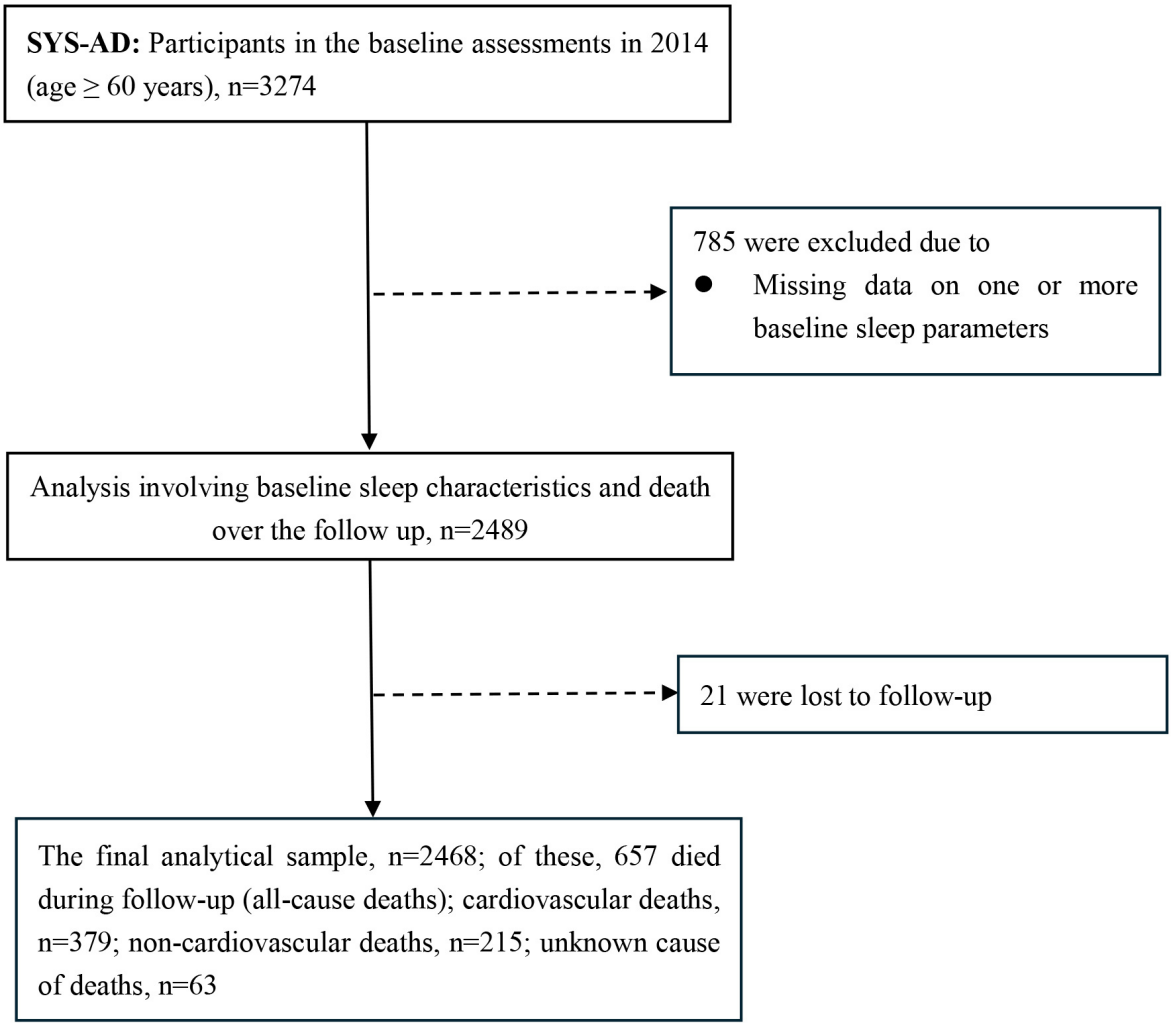
Flow chart of the study participants in SYS-AD, 2014–2022. SYS-AD, Shandong Yanggu Study of Aging and Dementia.

This cohort study included participants from the Shandong Yanggu Study of Aging and Dementia (SYS-AD) that targeted rural residents who were aged ≥60 years and living in Yanggu County, western Shandong Province, as previously reported.^15 16^ In brief, at baseline (August–December 2014), 3274 participants completed face-to-face interviews, clinical examinations and laboratory tests. Out of these, 785 were excluded due to missing information related to sleep at baseline and 21 were excluded due to loss to follow-up. The end of follow-up was death or 31 December 2022, whichever came first. Thus, the final analytical sample included 2468 participants. Figure 1 provides the flow chart of the study participants.

### Data collection

At baseline, trained research staff collected data via face-to-face interviews, clinical examinations, neuropsychological tests and laboratory tests following standard procedures, as previously reported.^17^ In brief, we collected data on socio-demographic factors (eg, age, sex and education), lifestyles (eg, smoking and alcohol consumption), health conditions (eg, hypertension and diabetes) and use of medications (eg, antihypertensives, lipid-lowering agents and hypnotics). Weight and height were measured in light clothes without shoes. Arterial blood pressure was measured on the right upper arm in a seated position using an electronic sphygmomanometer after a 5 min rest. After an overnight fast, peripheral blood samples were taken, and fasting blood glucose and lipids were measured at the clinical laboratory of local town hospital.

### Assessments of sleep characteristics

The sleep characteristics of participants at baseline were assessed using the Pittsburgh Sleep Quality Index (PSQI), a 19-item self-reported questionnaire designed to assess sleep quality over the last month. The PSQI comprises seven components: subjective sleep quality, sleep latency, sleep duration, habitual sleep efficiency, sleep disturbances, use of sleeping medication and daytime dysfunction.^18^ The total PSQI score ranges from 0 to 21, with a higher score indicating worse sleep quality. Poor sleep quality was defined as a total PSQI score >5.^19^ Sleep duration was assessed with the question: “During the past month, how many hours of actual sleep did you get at night?” Self-reported bedtime and risetime were used to assess TIB and mid-sleep time. TIB was calculated as the interval between bedtime and risetime, while mid-sleep time was defined as the halfway point between the two timepoints. Sleep latency was referred to as time (minutes) spent awake in bed before falling asleep, and long sleep latency was defined as the latency >30 min. Sleep efficiency was calculated by dividing the number of hours of sleep by the number of hours spent in bed, and low sleep efficiency was defined as sleep efficiency ≤85%.^20^

We categorised participants into tertiles based on the distribution of their sleep parameters, in line with the previous publication.^17^ In addition, we used restricted cubic splines (RCS) to explore their potential non-linear associations with all-cause mortality. If the RCS curve showed non-linear associations with mortality, we used the medium tertile of sleep parameters as the reference group. Otherwise, we considered the upper or lower tertile as the reference group. Thus, sleep duration (hours) was divided into short (<7), normal (7–8, reference) and long (>8); nocturnal TIB (hours) as short (<8, reference), normal (8–9) and long (>9); bedtime as early (before 21:00), middle (21:00–22:00) and late (22:00 or later, reference); and mid-sleep time as early (before 01:00), middle (01:00–01:30, reference) and late (after 01:30).

### Ascertainment of outcomes at follow-up

The vital status until 31 December 2022 and the causes of death for all participants were ascertained via linkage to death records or interviews with the village doctors or both. We chose 31 December 2022 as the end of follow-up because the dynamic zero-COVID-19 policy ended in early January 2023 in China.^21^ The study outcomes included all-cause mortality, cardiovascular mortality (eg, death due to acute myocardial infarction, pulmonary heart disease, intracerebral haemorrhage, cerebral infarction and non-traumatic intracranial haemorrhage) and non-cardiovascular mortality.

### Assessments of covariates

We considered demographic factors, lifestyles and health conditions as covariates in the analysis. Education was classified into no formal school education, primary school and middle school and above. Body mass index (BMI) was calculated as weight in kilograms divided by height squared in metres (kg/m^2^) and was categorised as normal weight (<24.0 kg/m^2^), overweight (24.0–27.9 kg/m^2^) and obese (≥28 kg/m^2^). Smoking and alcohol consumption were divided into never or ever smoking or drinking alcohol. Hypertension was defined as arterial blood pressure≥140/90 mm Hg or current use of any antihypertensive drugs.^22^ Diabetes was defined as fasting blood glucose≥7.0 mmol/L or current use of antidiabetic drugs or self-reported history of diabetes made by a physician.^23^ Dyslipidaemia was defined as total cholesterol≥6.2 mmol/L or triglyceride≥2.3 mmol/L or low-density lipoprotein cholesterol≥4.1 mmol/L or high-density lipoprotein cholesterol<1.0 mmol/L or current use of hypolipidaemic drugs.^24^

### Statistical analysis

The follow-up time was estimated from the date of baseline interviews to the date of death or 31 December 2022 (censored). The adjusted Kaplan-Meier plots were used to evaluate the overall survival by different sleep characteristics (eg, sleep duration, TIB, bedtime and mid-sleep time), and the survival curves were compared with the log-rank test. The association between sleep characteristics at baseline and all-cause, cardiovascular and non-cardiovascular mortality determined over the follow-up periods was assessed through Cox proportional-hazards models. The proportional-hazard assumption was verified using the Schoenfeld residual method. We adjusted for age, sex and education in model 1 and additionally adjusted for BMI, alcohol consumption, smoking, use of hypnotics, hypertension, diabetes and dyslipidaemia in model 2.

Furthermore, RCS Cox regression was applied to assessing the dose–response association of self-reported sleep characteristics with all-cause, cardiovascular and non-cardiovascular mortality, with 3 knots at 10th, 50th and 90th percentiles.

R platform (R Foundation for Statistical Computing; Vienna, Austria. https://www.R-project.org/) was used for all data analyses. Two-sided p<0.05 was considered statistically significant.

### Patient and public involvement

No patients or members of the public were involved in the study.

## Results

### Baseline characteristics of study participants

At baseline, the mean age of the 2468 participants was 71.45 (SD 5.91) years, 73.5% were aged 60–74 years, 57.3% were women and 43.2% had no formal schooling (table 1). During the median follow-up of 7.36 years, 657 (26.6%) participants died, including 379 (15.4%) cardiovascular deaths, 215 (8.7%) non-cardiovascular deaths and 63 deaths with unknown reasons.

**Table 1:** Characteristics of study participants at baseline (n=2468)

Characteristics	Participants
Age (years), mean (SD)	71.45 (5.91)
Age groups, n (%)	
60–74 years	1813 (73.5)
≥75 years	655 (26.5)
Women, n (%)	1413 (57.3)
Educational level, n (%)	
Illiteracy (no schooling education)	1067 (43.2)
Primary school	1018 (41.2)
Middle school and above	383 (15.5)
Body mass index (kg/m^2^), n (%)*	
<24	834 (43.1)
24–27.9	726 (37.5)
≥28.0	374 (19.3)
Alcohol consumption, n (%)	859 (34.9)
Ever smoking, n (%)	895 (36.4)
Hypertension, n (%)	1640 (66.7)
Diabetes, n (%)	328 (13.3)
Dyslipidaemia, n (%)	584 (23.7)
Hypnotics, n (%)	181 (7.3)
Sleep duration (hours), mean (SD)	7.28 (1.64)
Time in bed (hours), mean (SD)	8.44 (1.26)
Bedtime (hours:min), mean (SD)	21:06 (00:56)
Mid-sleep time (hours:min), mean (SD)	01:18 (00:36)
Sleep efficiency (%), mean (SD)	86.42 (16.4)
Pittsburgh Sleep Quality Index score, mean (SD)	5.43 (3.86)

* Numbers of participants with missing values were 534 for body mass index. In subsequent analyses, a dummy variable was created to represent the group of participants with missing values.

### Associations of baseline sleep characteristics with all-cause mortality (n=2468)

**Figure 2:**
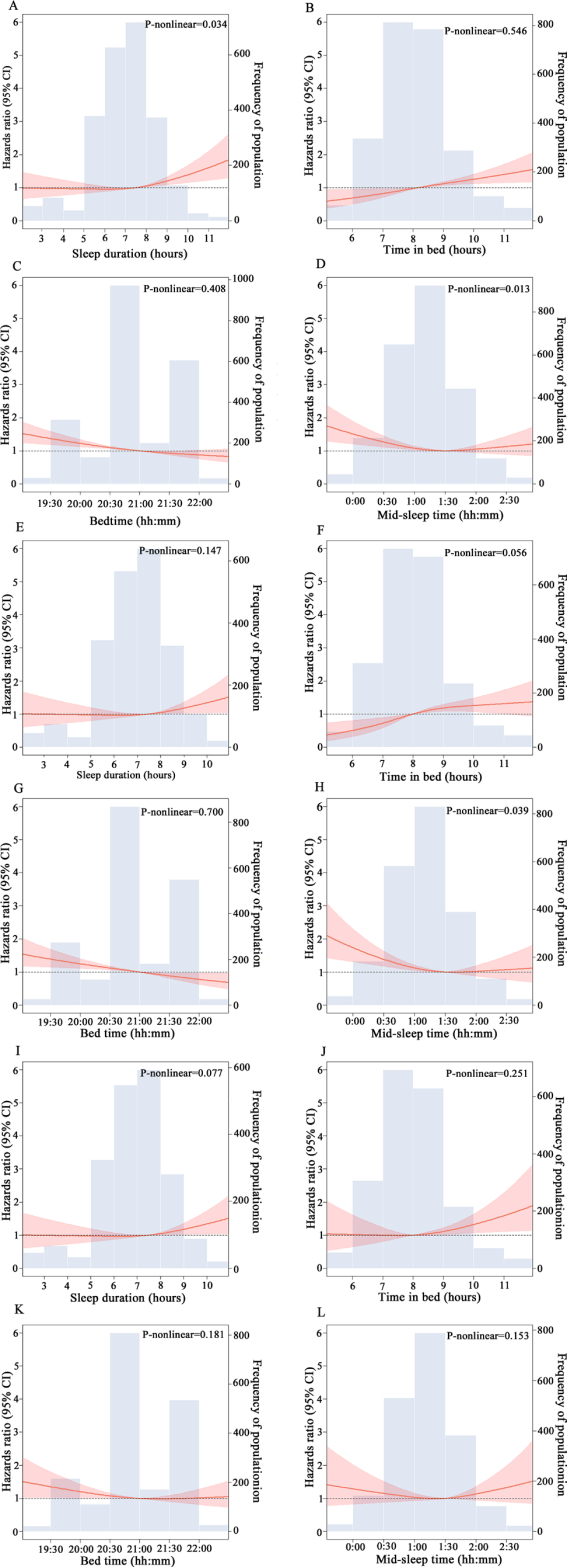
Multivariable-adjusted spline curves for associations of sleep characteristics with all-cause mortality (A–D), cardiovascular mortality (E–H) and non-cardiovascular mortality (I–L). Solid lines represented HRs of all-cause mortality, adjusting for age, sex, education, body mass index, alcohol consumption, smoking, hypnotics, hypertension, diabetes and dyslipidaemia.

Kaplan-Meier analysis showed lower overall survival among participants with long sleep duration, long TIB, early bedtime and early mid-sleep time (online supplemental figure S1A–D). Adjusting for multiple confounders, RCS curves showed a J-shaped or a reverse J-shaped association of all-cause mortality with sleep duration and mid-sleep time, respectively (figure 2). Early mid-sleep time was significantly associated with all-cause mortality. TIB and bedtime exhibited a linear relationship with all-cause mortality. As a continuous variable, per 1-hour increase in sleep duration was associated with an 8% increased risk of all-cause mortality (95% CI 1.02 to 1.15), every 1-hour delay in TIB was associated with a 14% increased risk of all-cause mortality (95% CI 1.08 to 1.21) and every 1-hour delay in bedtime was associated with a 17% reduced risk of all-cause mortality (95% CI 0.77 to 0.90).

When sleep characteristics were divided into tertiles, long sleep duration, long TIB, early bedtime and early mid-sleep time at baseline were significantly associated with an increased risk of all-cause mortality (table 2). There were no significant associations of sleep quality, sleep latency and sleep efficiency with all-cause mortality.

**Table 2:** Associations of baseline sleep characteristics with all-cause mortality, cardiovascular and non-cardiovascular mortality (n=2468)

Baseline sleep characteristics	All-cause mortality		Cardiovascular mortality		Non-cardiovascular mortality	
	n/N	HR (95% CI)	n/N	HR (95% CI)	n/N	HR (95% CI)
Sleep duration (hours)						
<7	166/698	0.98 (0.80 to 1.19)	97/629	1.00 (0.78 to 1.30)	61/593	1.05 (0.76 to 1.46)
7–8	286/1221	1.00 (reference)	160/1095	1.00 (reference)	98/1033	1.00 (reference)
>8	205/549	1.27 (1.06 to 1.53)*	122/466	1.27 (1.00 to 1.63)	56/400	1.23 (0.88 to 1.72)
Time in bed (hours)						
<8	108/597	1.00 (reference)	55/544	1.00 (reference)	44/533	1.00 (reference)
8–9	361/1418	1.20 (0.97 to 1.50)	222/1279	1.43 (1.06 to 1.93)*	112/1169	1.01 (0.71 to 1.44)
>9	188/453	1.63 (1.27 to 2.08)***	102/367	1.61 (1.15 to 2.27)**	59/324	1.64 (1.09 to 2.47)*
Bedtime						
22:00 or later	21/112	1.00 (reference)	10/101	1.00 (reference)	11/102	1.00 (reference)
21:00–22:00	401/1757	1.13 (0.73 to 1.76)	224/1580	1.35 (0.71 to 2.56)	144/1500	0.81 (0.44 to 1.51)
Earlier than 21:00	235/599	1.58 (1.00 to 2.49)*	145/509	1.94 (1.01 to 3.72)*	60/424	0.98 (0.51 to 1.89)
Mid-sleep time						
Earlier than 01:00	152/398	1.45 (1.20 to 1.76)***	100/346	1.69 (1.32-2.15)***	37/283	1.21 (0.84 to 1.76)
01:00–01:30	362/1470	1.00 (reference)	206/1314	1.00 (reference)	123/1231	1.00 (reference)
Later than 01:30	143/600	1.03 (0.85 to 1.25)	73/530	0.95 (0.73 to 1.25)	55/512	1.13 (0.82 to 1.56)
Sleep quality						
Good	395/1476	1.00 (reference)	221/1081	1.00 (reference)	128/1209	1.00 (reference)
Poor	262/992	1.00 (0.85 to 1.19)	158/888	1.09 (0.88 to 1.36)	87/817	1.01 (0.75 to 1.35)
Sleep latency (min)						
≤30	206/842	1.00 (reference)	116/752	1.00 (reference)	70/706	1.00 (reference)
>30	451/1626	1.10 (0.93 to 1.30)	263/1438	1.11 (0.89 to 1.39)	145/1320	1.12 (0.84 to 1.49)
Sleep efficiency (%)						
>85	447/1712	1.00 (reference)	256/1521	1.00 (reference)	142/1407	1.00 (reference)
≤85	210/756	1.01 (0.85 to 1.19)	123/669	1.02 (0.82 to 1.28)	73/619	1.09 (0.81 to 1.46)

n/N indicates the number of death/number of study participants.

The HRs (95% CIs) were derived from the models that were adjusted for age, sex, education, body mass index, alcohol consumption, smoking, use of hypnotics, hypertension, diabetes and dyslipidaemia.

*p<0.05, **p<0.01, ***p<0.001.

### Associations of baseline sleep characteristics with cardiovascular (n=2190) or non-cardiovascular mortality (n=2026)

Kaplan-Meier analysis showed lower cardiovascular survival among participants with long sleep duration, long TIB, early bedtime and early mid-sleep time (online supplemental figure S1E–H). Adjusting for multiple confounders, RCS curves showed a reverse J-shaped association between mid-sleep time and cardiovascular mortality (figure 2).

When sleep parameters were divided into tertiles, long sleep duration, extended TIB, early bedtime and early mid-sleep time at baseline were significantly associated with an increased risk of cardiovascular mortality (table 2). We found no significant associations of sleep quality, sleep latency and sleep efficiency with cardiovascular mortality. Of all the examined sleep parameters, only long TIB was associated with an increased risk of non-cardiovascular mortality (online supplemental figure S1I–L).

## Discussion

In this population-based cohort study targeting rural-dwelling older adults in China, the RCS analysis showed a J-shaped association of sleep duration with all-cause mortality, and a reverse J-shaped association of mid-sleep time with all-cause and cardiovascular mortality. Furthermore, long sleep duration and early sleep timing might be associated with increased risks of both all-cause and cardiovascular mortality, and long TIB might be linked to an increased risk of all-cause, cardiovascular and non-cardiovascular mortality.

Population-based cohort studies have so far yielded mixed results regarding the association between sleep duration and all-cause mortality.^910 14 25^,^28^ A meta-analysis of cohort studies found that a long sleep duration was associated with an increased risk of mortality,^29^ which was in line with the results from our study. The US Sleep Heart Health Study of people aged ≥40 years observed a J-shaped association of self-reported sleep duration with all-cause and cardiovascular mortality.^30^ We also found that a long sleep duration was associated with an increased risk of cardiovascular mortality, which could potentially reflect the underlying health conditions that may contribute to CVD and death. Of note, we found no evidence for the association of short sleep duration with either all-cause or cardiovascular mortality. These discrepancies may be partly explained by the differences in the operational definitions of short sleep duration across studies. For instance, the association with mortality tends to be stronger in studies that define short sleep duration as ≤5 hours per day.^31 32^ While the prospective Shanghai study of middle-aged and older adults showed the associations of sleep duration with CVD and all-cause mortality, reporting modest but statistically non-significant increases in risk associated with short sleep duration (eg, 4–5 hours),^25^ which is generally consistent with our study. In addition, heterogeneity in the demographic characteristics of study populations and adjustment for potential confounders may also partially explain the discrepant findings across studies.

Population-based studies that explore the relationships between TIB and sleep timing with mortality are scarce. The US Women’s Health Initiative study showed that TIB was a stronger risk factor for mortality than total sleep time.^33^ Indeed, spending excessive TIB can elicit daytime lethargy and exacerbate sleep fragmentation, resulting in a vicious cycle of further TIB and further sleep fragmentation.^34^ Moreover, pooling analysis of three cohorts using machine-learning approach revealed that TIB was one of the most predictive individual sleep characteristics for all-cause and cardiovascular mortality.^35^ We found that extended TIB was associated with an increased risk of all-cause, cardiovascular and non-cardiovascular mortality. Our study suggested that older adults who went to bed earlier than 21:00 had an increased risk of all-cause or cardiovascular mortality. This is in contrast with findings from the large-scale Prospective Urban Rural Epidemiology study in 21 countries, which showed that bedtime 22:00 or earlier (earlier sleepers) and 00:00 or later (late sleepers) were associated with an 11% and 18% higher risk of all-cause mortality, respectively.^36^ Chronotype (eg, morningness or eveningness) could be related to disrupted circadian rhythms. Population-based cohort studies have indicated that morning chronotype is linked with an increased risk of hypertension and diabetes,^37 38^ which could contribute to an elevated risk of mortality. Nevertheless, previous studies have rarely investigated the association between mid-sleep time and mortality. We discovered a reverse J-shaped association between mid-sleep time and all-cause mortality. This is in contrast with leveraging wearable data from the UK Biobank cohort of middle-aged adults (age 40–69 years), which showed that early (before 02:30) or late (after 03:30) mid-sleep time was associated with an increased mortality risk.^39^ Compared with Western populations and urban residents, rural older adults in China usually have a morningness chronotype.

Although the mechanisms underlying the relationship between sleep problems and mortality remain unclear, several possible explanations have been proposed. Long sleep duration has been associated with immune function,^40^ sleep fragmentation and fatigue^41^ or chronic diseases (eg, cognitive impairment,^17 42^ depression and diabetes).^43^ Having a morning chronotype could indicate a higher likelihood of experiencing health issues that may increase the risk of death or CVD. The mechanisms underlying the association of advanced sleep timing with adverse health events are not fully understood, which deserves further exploration.

Our population-based study engaged rural-dwelling older adults in China where a considerable proportion of people had no or very limited formal education and relatively low socio-economic status. Moreover, we evaluated multiple sleep characteristics, including the timing of sleep, which serves as an indicator of circadian rhythms. Nevertheless, several limitations of this study deserve discussion. First, sleep characteristics were assessed through self-reported data; thus, recall bias may influence the association. Second, we examined only baseline sleep characteristics in relation to mortality, and changes in sleep habits or patterns during the follow-up period could bring about non-differential misclassification of sleep characteristics, which might affect the estimation of their true associations. Third, despite the fact that a wide range of confounding factors had been taken into account in the analysis, the possibility of residual confounding cannot be completely ruled out (eg, sleep apnoea insomnia and depressive symptoms). Fourth, participants in this study were recruited from only one rural region in western Shandong Province, which should be kept in mind when generalising the research findings to other populations. Finally, due to the observational nature of this study, caution is needed when interpreting the observational associations as possible causal relationships.

In conclusion, our study shows that long sleep duration and early sleep timing may be associated with all-cause and cardiovascular mortality, and that long TIB may be linked to an increased risk of all-cause, cardiovascular and non-cardiovascular mortality in Chinese rural older adults. These findings provide important insights into the complex relationships between sleep characteristics and all-cause and cause-specific mortality among rural older adults in China. Future intervention studies are imperative to clarify whether pharmacological and non-pharmacological interventions to optimise sleep conditions could help improve survival in older adults.

## Supplementary material

10.1136/bmjopen-2024-094928 online supplemental file 1

## Data Availability

Data are available upon reasonable request.
