# Supplementary material for: Associations of sleep characteristics with all-cause, cardiovascular and non-cardiovascular mortality among rural Chinese older adults: a cohort study
Source: BMJ Open. 2025 Jun 22;15(6):e094928. doi: 10.1136/bmjopen-2024-094928 (PMC12184377; doi:10.1136/bmjopen-2024-094928)
Supplement: online supplemental file 1 [file bmjopen-15-6-s001.docx]

**Supplementary materials**

Associations of sleep characteristics with all-cause, cardiovascular, and non-cardiovascular mortality among rural Chinese older adults: A cohort study

Lu J, et al.

**Contents**

**Supplementary Figure S1.** Adjusted Kaplan–Meier curves and 95% confidence intervals (shaded areas) of overall survival for all-cause (panel a-d), cardiovascular (panel e-h), and non-cardiovascular (panel i-l) mortality by levels of sleep parameters.


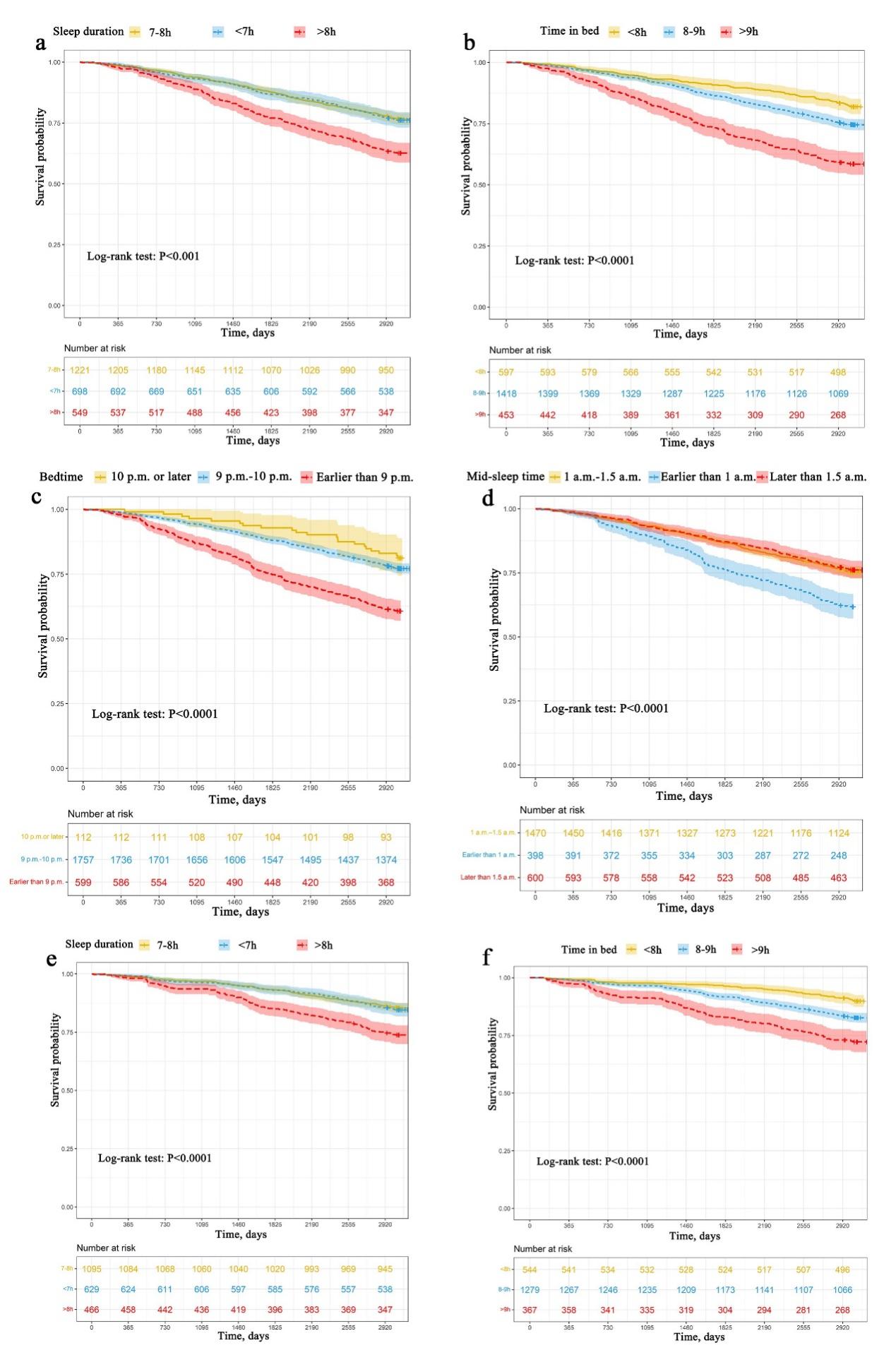


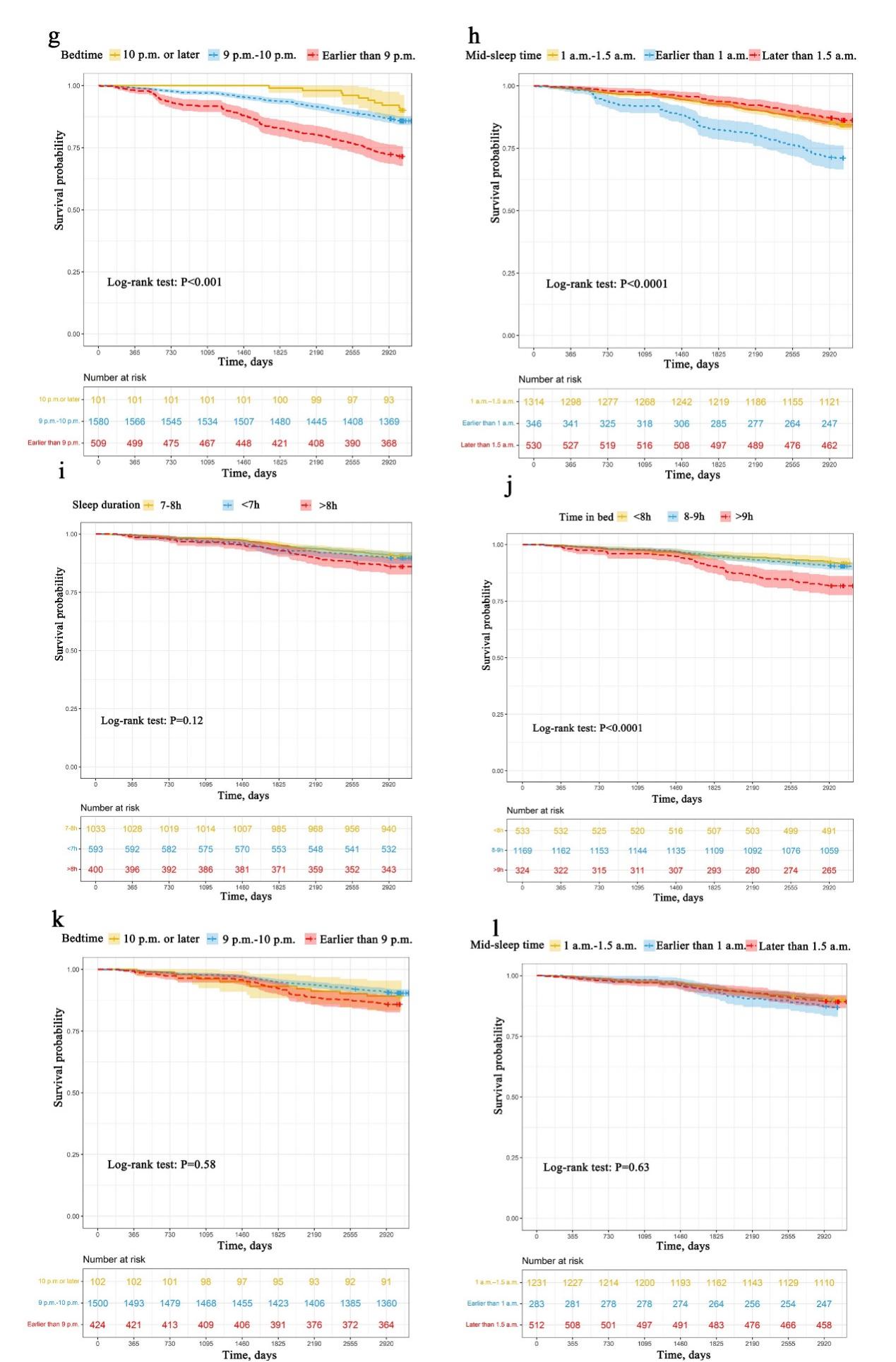


**Supplementary Figure S1**. Adjusted Kaplan-Meier curves and 95% confidence intervals (shaded areas) of overall survival for all-cause (panel a-d), cardiovascular (panel e-h), and non-cardiovascular (panel i-l) mortality by levels of sleep parameters.
