# Supplementary material for: Associations of sleep characteristics with all-cause, cardiovascular and non-cardiovascular mortality among rural Chinese older adults: a cohort study
Source: BMJ Open. 2025 Jun 22;15(6):e094928. doi: 10.1136/bmjopen-2024-094928 (PMC12184377; doi:10.1136/bmjopen-2024-094928)
Supplement: Supplementary file 2 [file bmjopen-2024-094928.reviewer_comments.pdf]

PEER REVIEW HISTORY

BMJ Open publishes all reviews undertaken for accepted manuscripts. Reviewers are asked to complete a checklist review form and are provided with free text boxes to elaborate on their assessment. These free text comments are reproduced below.

ARTICLE DETAILS

Title (Provisional)

Associations of sleep characteristics with all-cause, cardiovascular, and non-cardiovascular mortality among rural Chinese older adults: A cohort study

Authors

Lu, Jie; Liu, Rui; Wang, Jiafeng; Liu, Keke; Ren, Juan; Zhao, Tong; Ren, Yifei; Mao, Ming; Liang, Yajun; Tang, Shi; Du, Yifeng; Qiu, Chengxuan

---

VERSION 1 - REVIEW

**Reviewer**	**1**
**Name**	**Healy , William J**
**Affiliation**	**Division of Pulmonary, Critical Care, and Sleep Medicine Medical College of Georgia at Augusta University Augusta, Augusta, GA, USA.**
**Date**	**14-Dec-2024**
**COI**	**None**

This is an exciting population-based study of 2468 rural Chinese adults that leverages questionnaire sleep data to make inferences regarding sleep duration and timing with all-cause and cardiovascular mortality. My primary criticism is that the language of long sleep duration and early sleep timing is said to be associated with increased mortality. Instead, perhaps we could say it "may" be associated with these things-especially given this study design we cannot more definitively say that in my opinion.

**Reviewer**	**2**
**Name**	**Kusunoki , Yoshiki**
**Affiliation**	**Hyogo Medical University**
**Date**	**13-Apr-2025**
**COI**	**None**

In a population-based cohort study, the authors investigated the association between sleep timing and time in bed (TIB) and all-cause mortality, cardiovascular mortality, and other

outcomes among older adults living in rural areas. The results of this study indicated that long sleep duration, early sleep timing, and long TIB are associated with all-cause and cardiovascular mortality in rural Chinese elderly.

Various studies have shown an association between sleep duration and cardiovascular death, so the novelty of this study may be lacking. On the other hand, the study is very well organized and includes some interesting points, such as the reference to the risk of TIB.

Let me offer a few comments on this paper.

Longer sleep duration and earlier sleep timing were shown to be associated with an increased risk of both all-cause and cardiovascular mortality. The authors note that morning chronotypes are more common in rural populations. If it is possible, the authors should also investigate the association between waking time and all-cause mortality among their subjects.

Did the authors find no association between short sleep duration and cardiovascular disease from this review? It would be good to have a discussion on this point as well.

VERSION 1 - AUTHOR RESPONSE

Reviewer: 1

Dr. William J Healy, Division of Pulmonary, Critical Care, and Sleep Medicine Medical College of Georgia at Augusta University Augusta, Augusta, GA, USA.

Comments to the Author:

This is an exciting population-based study of 2468 rural Chinese adults that leverages questionnaire sleep data to make inferences regarding sleep duration and timing with all-cause and cardiovascular mortality. My primary criticism is that the language of long sleep duration and early sleep timing is said to be associated with increased mortality. Instead, perhaps we could say it "may" be associated with these things-especially given this study design we cannot more definitively say that in my opinion.

Response: We thank the reviewer for the valuable comments that we totally agree with. Indeed, as an observational study, we are not able to definitely claim their temporal relationship. Thus, we have now rephrased the sentence throughout the manuscript wherever necessary, as our reviewer suggested *(see page 2-3, lines 43-45; page 11, lines 221-222)*.

Reviewer: 2

Dr. Yoshiki Kusunoki , Hyogo Medical University

Comments to the Author:

In a population-based cohort study, the authors investigated the association between sleep timing and time in bed (TIB) and all-cause mortality, cardiovascular mortality, and other outcomes among older adults living in rural areas. The results of this study indicated that long sleep duration, early sleep timing, and long TIB are associated with all-cause and

cardiovascular mortality in rural Chinese elderly.

Various studies have shown an association between sleep duration and cardiovascular death, so the novelty of this study may be lacking. On the other hand, the study is very well organized and includes some interesting points, such as the reference to the risk of TIB.
Response: We thank the reviewer for the kind summary of our work, along with valuable suggestions below that help us to improve our work.

Let me offer **a few comments** on this paper.

Longer sleep duration and earlier sleep timing were shown to be associated with an increased risk of both all-cause and cardiovascular mortality. The authors note that morning chronotypes are more common in rural populations. If it is possible, the authors should also investigate the association between waking time and all-cause mortality among their subjects.

Response: We thank the reviewer for the valuable comments, which are very important. We carefully addressed these comments as described below. Indeed, waking time is an important component of sleep timing. In response to the reviewer suggestion, we analyzed the associations between waking time and all-cause, cardiovascular, and non-cardiovascular mortality, and we found no significant associations between them. We reported these results only in this response letter in Table 1 below, but we are open to report the full results from these analyses in the main text of the manuscript (e.g., methods, results, and discussion sections) if the reviewer requests.

Table 1. Associations of baseline waking time with all-cause mortality, cardiovascular and non-cardiovascular mortality (n=2468)

**Waking time**	**All-cause mortality**		**Cardiovascular mortality**		**Non-Cardiovascular mortality**	
	**n/N**	**HR (95% CI)**	**n/N**	**HR (95% CI)**	**n/N**	**HR (95% CI)**
Earlier than 5 a.m.	46/178	1.00 (reference)	29/161	1.00 (reference)	12/144	1.00 (reference)
5 a.m.–6 a.m.	519/2027	1.05 (0.78-1.43)	294/1802	0.99 (0.67-1.45)	178/1686	1.43 (0.79-2.60)
Later than 6 a.m.	92/263	1.18 (0.82-1.69)	56/227	1.11 (0.70-1.75)	25/196	1.43 (0.71-2.88)

Abbreviations: HR, hazard ratio; CI, confidence interval.

n/N indicates the number of deaths/number of study participants.

The hazard ratios (95% confidence intervals) were derived from the models that were adjusted for age, sex, education, body mass index, alcohol consumption, smoking, use of hypnotics, hypertension, diabetes, and dyslipidemia.

Did the authors find no association between short sleep duration and cardiovascular disease from this review? It would be good to have a discussion on this point as well.

Response: We thank the reviewer for the valuable comment. In response, we briefly discussed the results on the association between short sleep duration and cardiovascular disease in the Discussion section, and provided possible explanations to the lack of association between a short sleep duration and cardiovascular mortality in our study *(see page 11-12, lines 229-239)*.
